# Subjective well-being of current and former elite athletes in the German armed forces

**DOI:** 10.3389/fspor.2024.1256103

**Published:** 2024-01-26

**Authors:** Peter Ehnold, Franziska Vollmann, Torsten Schlesinger

**Affiliations:** ^1^ Department of Sport & Management, IST-University of Applied Sciences Düsseldorf, Düsseldorf, Germany; ^2^ Department of Sport Management, Faculty of Sport Science, Ruhr-University Bochum, Bochum, Germany; ^3^ Institute of Human Movement Science and Health, Chemnitz University of Technology, Chemnitz, Germany

**Keywords:** German military’s sports promotion group, subjective well-being, current and former German elite athletes, influencing factors, linear regression models

## Abstract

**Introduction:**

The German military's sports promotion group (SPG) is an entity that aims to minimize the risks inherent in elite sports careers. Risks that manifest themselves, for example, in a lack of financial resources or failed dual careers could undermine the subjective well-being (SWB) of athletes, which can have a negative impact on athletic performance. A differentiated understanding of factors that influence SWB is therefore necessary.

**Methods:**

Within the scope of a survey, a sample of *n* = 414 current and *n* = 298 former sports soldiers was generated. Linear regression models (blockwise) were used to analyze the influence of the SPG framework conditions, the influence of top sport-specific factors, and the influence of socio-demographic/socio-economic factors on the SWB of current and former sport soldiers.

**Results:**

Current and former sports soldiers showed high satisfaction scores and were also optimistic about the future. Among current sports soldiers, men had higher SWB values. In addition, satisfaction with SPG services, satisfaction with their athletic careers, and satisfaction with their current income all influenced the athletes' SWB. Among former sports soldiers, persons in a stable partnership had higher SWB values. Furthermore, satisfaction with SPG services, a career end that was perceived as involuntary and satisfaction with current income were relevant for SWB in this cohort.

**Discussion:**

This study addresses the call for a more athlete-centered approach in sports science research and the findings have several implications for organized sports to promote SWB in elite athletes.

## 1 Introduction

Careers in elite sport are risky, as athletic success is something scarce and thus highly uncertain (1). There are also risks with regard to physical performance and health and the associated duration of athletic careers (2–6) in the context of enabling dual careers (7–11) and in relation to financial resources (12, 13). Politicians also recognize the risks of elite sports careers, which leads to government institutions becoming involved in the promotion of elite sports. Support in government-related sectors is of great importance, with the military occupying a prominent position in many countries (14). Top-level sports promotion in the military can be observed in various countries [e.g., Austria (15); Switzerland (16); Great Britain (17); Italy (14); USA (14)], although there are sometimes considerable differences between the specific promotion structures in the individual countries. In Germany in particular, the promotion of elite sports in the military is of particular importance, as the military's sports promotion group (SPG) provides approximately 850 positions for elite athletes (18).

The specific physical, psychological, and economic risks of elite sports careers, in combination with other risks that can also be relevant for non-elite athletes, can endanger athletes' well-being (19). Athlete well-being is important for organized sports, as it is a crucial factor influencing athletic performance (20, 21). There is also a risk that careers in elite sports are terminated prematurely due to dissatisfaction (distress, anxiety, and depression) despite existing athletic prospects (drop-out as a coping strategy, 8). In addition, as cases of violence and abuse in elite sports become known (22, 23), the demands on organized sports to better protect athletes and attach greater importance to their well-being are increasing.

Given the importance of athlete well-being, a nuanced understanding of factors that influence well-being is required. By directly addressing both facilitating and inhibiting factors, organized sports can effectively target well-being (24). This is particularly true of elite sports promotion within the military sector, given the great significance of elite sports promotion in the military in many countries and the special possibilities for direct and indirect influence on athletes and structural conditions due to the links to the government. This article builds on this and explores the central questions: *How do current and former German sports soldiers assess their general well-being and what factors influence it?*

### 1.1 Research context—elite sports promotion in the German armed forces

In order to make it easier to combine vocational training or a job with an elite sporting career, the German state funds approximately 1,450 state positions in the German Armed Forces, German Police, German Customs Administration, police services in the federal states and the fire brigades (25). The largest proportion of these are in the German Armed Forces, which currently funds around 850 positions for both Olympic and non-Olympic elite athletes in 15 SPGs in different locations. Therefore, the SPGs are the central funding body for dual careers for elite athletes in Germany (26). The aim of elite sports funding in the German Armed Forces is to “guarantee the compatibility of elite sport and a career as part of (dual) career planning” (27, p. 39). The mandate for establishing the SPGs dates back to a 1968 resolution from the German Federal Parliament. State support for elite sport in Germany is provided on the basis of the “federal government's interest in an appropriate representation of the Federal Republic of Germany at home and abroad” (28, p. 19). In addition, elite sport should promote integration and solidarity, communicate values through a role model function, contribute to international understanding and help to enhance the reputation of Germany abroad (29).

Certain requirements apply for athletes to be accepted into the German Armed Forces elite sports promotion program (30). In addition to the willingness to become a soldier and the approval of the respective national sports federations and the German Olympic Sports Confederation (DOSB), the positions for sports soldiers are usually filled exclusively with Olympic and prospective squad athletes.

After four weeks of basic training in the SPG, the athletes are initially deployed as military service volunteers and later as regular soldiers. An essential incentive in the SPG is to largely replace military content with sports content (duty roster = training time). This creates extensive freedom to train and participate in competitions, or to complete vocational training. The sports soldiers receive a regular salary corresponding to their military rank and are covered by health and social insurance, but in return, they have to perform few, if any, military duties. They also do not have to be available for military operations overseas (conflicts). Rather, the athletes are “expected” to achieve elite sporting performance and success in international competitions. Prize money, sponsorship income or similar are not offset against the military salary.

The German Armed Forces Vocational Support Services (VSS) plays a central role in supporting the compatibility of an athletic career and vocational training (31). The VSS offers specific opportunities for the realization of dual careers, i.e., in addition to a career in elite sports, vocational training and further education (studies, acquisition of job-related qualifications and degrees) are also supported. For sports soldiers, the supporting measures have been made largely flexible, so that a dual career can also be pursued while they are still members of the SPG. Thus, professional training or studies that accompany the period of service and are appropriate for competitive sports are possible during active duty in the SPG, with individually adapted attendance phases within the framework of available time (32). Within the German Armed Forces, there are selected training opportunities available to sports soldiers (for example, career-oriented continuing education and internships) that can be completed simultaneously with elite sports. The temporal and financial scope of support generally depends on the duration of the commitment to the SPG (31).

### 1.2 Literature review

Despite the great importance of military funding agencies for elite sports, the well-being of current or former sports soldiers has not been the subject of research so far. Nevertheless, a number of studies are available that analyze the well-being of current or former elite athletes or of elite athletes who were beginning the transition phase into their post-sporting career. The existing literature focuses on the influence of psychological factors on well-being, especially in the case of active elite athletes The self-determination and achievement of goals (competitive assessments) within a specified amount of time (e.g., 33–37) prove to be particularly relevant. The ability to adopt value-oriented behaviors and engage in functioning relationships (21), and the factors mindfulness and self-esteem (38) are positively associated with well-being. In addition to psychological factors, available supportive structures and services (39) are important. Regarding socioeconomic factors, a negative influence of age and poor health and a positive effect of income have been found (13, 40). Central factors that influence the well-being of former elite athletes are, for example, the type of sport (41, 42), the age that participants began their sport (43), the level of athletic performance (42), or the reason for retirement from elite sports (37, 40). During the transition phase, the form of retirement (voluntary or involuntary), the personal approach to retirement, and any coping strategies and new goals for life after an athletic career are relevant to well-being (e.g., 44–46). It has also been shown that well-being is subject to fluctuations over time during the transition phase to a post-sports career, first decreasing after retirement and then increasing again (47, 48).

The objective of this study is to expand on previous studies that focused primarily on psychological factors and, in particular, to analyze the possible direct or indirect influence of the SPG support structures on the well-being of elite athletes. In addition, high-level athletic aspects and socio-demographic and socio-economic factors will be taken into account.

## 2 Theoretical framework

### 2.1 Conceptualization of SWB

Well-being is a complex construct. A basic distinction is made between eudaimonic and hedonic well-being (21). At the center of the eudaimonic approach are psychological well-being (49) and social well-being (50). Psychological well-being focuses on psychological aspects of living life in a fulfilling and deeply satisfying way by actualizing one's strengths and overcoming challenges (49). Six dimensions are considered central to a person's self-actualization: Self-acceptance, personality development, relationships with others, autonomy, coping with everyday life, and life goals (51). Social well-being results from perceived social support and integration, as well as friendships and social activities (52). Eudaimonic well-being is process-oriented rather than result-oriented and strives for lasting happiness through the realization of one's own potential. Hedonic well-being involves positive experiences, feeling good, and immediate sensory pleasure. The focus is on subjective well-being (SWB), which is divided into cognitive-evaluative and affective-emotional components (53). Within the cognitive dimension, a distinction is also made between general and domain-specific life satisfaction (54).

The eudaimonic and hedonic approaches differ significantly in some cases, but they also overlap (55). This paper primarily follows the hedonic approach. However, elite athletes are confronted with numerous challenges in their careers, and how they deal with them can have an impact on their life satisfaction. Therefore, eudaimonic facets were also included in the analysis (40). However, for the sake of clarity and simplification, only SWB will be referred to in the following.

When analyzing factors that influence SWB, a distinction is made between bottom-up and top-down factors (56, 57). The bottom-up approach focuses on external factors, such as general framework conditions (politics and environment) or specific life circumstances (in childhood) (58). In contrast, the top-down approach (58) follows the premise that SWB is not changed by specific life events but is determined by a person's personality traits (59). Holistic models try to combine both approaches (60), as it is assumed that there is an interaction between the factors lying within and outside a person, which is why both approaches are relevant for explaining SWB (61). Nevertheless, due to the complexity and range of possible influencing factors, empirical studies often only include individual factors in the analysis (principle of parsimony). Depending on the question, the object of the study, and the research perspective, the focus is placed more on external factors (framework conditions and life circumstances) or on internal factors (personality traits). The present study focuses on the structural framework of the SPG and sport-specific factors, as well as socio-demographic and socio-economic factors.

### 2.2 Factors associated with SWB of current and former sports soldiers

In the following, possible factors influencing the SWB of current and former sports soldiers are reflected theoretically. The majority of the factors appear to be equally relevant for current and former sports soldiers. However, there are also factors that are not yet significant for current sports soldiers and no longer significant for former sports soldiers, which should be clarified in the explanations. Factors associated with an elite sporting career are also included for the former sports soldiers. Consequently, the involvement in the SPG is also taken into consideration. The former sports soldiers have already ended their sporting career and are therefore no longer members of the SPG. However, during their time as athletes the former sports soldiers were highly involved in elite sport and the SPG. The elite sports system makes disproportionate demands on athletes in terms of the time, material and social dimensions (62), which is why it is possible to talk of their hyperinclusion in elite sport (63). Owing to the very high significance of elite sport and the SPG for the former sports soldiers over many years, it should be investigated whether there is a relation with the current SWB. It is known that previous significant life circumstances (also after childhood) can have a direct or indirect influence on the SWB (64), which is particularly the case for specific life events (65). In the context of elite sport this could be, for example, outstanding sporting success or a premature, involuntary career end due to injury.

Current and former sports soldiers are or have been confronted with specific *framework conditions of the SPG*. On the one hand, the SPG act as temporary employers for top athletes, and on the other hand, specific support structures and services are intended to facilitate dual careers, i.e., the compatibility of elite athletics and vocational training or higher education. Like other professional fields of work (66–68), it can be assumed that the degree of satisfaction with the SPG as an employer impacts SWB, whereby this should apply in particular to current sports soldiers, as they are still members of the SPG.

Current and former sports soldiers are or were highly involved in elite sports, which is why *elite sport-specific factors* must be included in the analysis. Athletic success is the primary goal within elite sports (69). If top athletes achieve their desired athletic goals, this should have a direct positive effect on SWB, as the individual level of aspiration is satisfied (35) and there is a positive individual return on investment (time and physical effort is reflected in athletic success). As success increases popularity and media attention, income can develop positively for specific sports (13), which means that athletic success can also have a positive indirect influence on SWB. It is also important to examine the extent to which the amount of time spent participating in a sport influences the SWB of current sports soldiers. Elite sports often become a full-time job (69), which can lead to conflicting goals and issues as the limited time budget for other areas of life are limited, for example, a dual career as an important precautionary strategy for the time after the end of the sports career (70). The SWB of current sports soldiers would therefore be expected to decrease as the amount of training increases. In the context of elite sports, it is also important to note that pain, discomfort, and injuries are an everyday phenomenon and go along with high risks and uncertainties (6, 71). Consequently, injuries or illnesses leading to interruptions in sports careers can be expected to have a negative impact on the SWB of current sports soldiers (72). The end of their career and the transition from an active sports career into a post-sports career are considered to be significant phases in the life of elite athletes (11, 73, 74). It has been shown that the feeling of having to end their career involuntary and prematurely can have a negative effect on SWB among athletes (41, 44). The extent to which former sports soldiers feel that they can themselves decide when to end their career is therefore to be included in the analysis.

Furthermore, *socio-demographic and socio-economic factors* shout should also be analyzed. Previous studies suggest that education-related factors have a positive influence on SWB (e.g., 75–79). By associating higher education with aspects such as self-actualization, self-confidence, self-esteem, and freedom and flexibility regarding future career prospects, the athlete's level of education can directly influence SWB in a positive way (80, 81). In addition, an indirect influence is also conceivable, in that a higher level of education is associated with higher income, better health, and greater confidence, which increases SWB (82). On the other hand, level of education can also have negative effects on SWB through a failure to fulfil professional expectations (83) or through pressure in academic competition (84). In addition to the level of education, it is also important to examine the extent to which employment status has an impact on SWB among former sports soldiers, since this reflects, not least, the professional reorientation after the end of the sports career and the successful transition to a post-sports career. Some studies indicate a positive influence of (fulltime) employment on SWB (75, 79, 85), while other studies show no influence (86, 87). Furthermore, higher income or satisfaction with income is also associated with higher SWB (e.g., 40, 75, 78, 79, 88, 89).

In addition, a possible influence of gender, age, relationship status, and children is controlled for. Women tend to have higher SWB values compared to men (75, 79, 87, 89–91), although opposite effects have also been found (78, 86). In active elite athletes, SWB has been shown to decrease with age (40). Previous studies have found that people in committed relationships report higher SWB than people who are not in committed relationships (e.g., 75, 78, 79, 88–90, 92). Having their own children seems to have more of a negative impact on SWB in younger people, whereas it tends to have either no influence or a positive influence on people aged over 40 (93, 94).

## 3 Method

### 3.1 Sample

A sample of current and former sports soldiers was generated through a full survey via an online questionnaire administered between January 16, 2020 to November 30, 2020. The questionnaire was therefore largely conducted during the COVID-19 pandemic. This should be taken into consideration when interpreting the results.

First, all sports soldiers were included who were currently members of the SPGs at the 14 locations surveyed and had therefore not yet ended their elite sports careers (*N* = 771). Former sports soldiers who had left a SPG within the past ten years were also surveyed (reference year 01/2010, *N* = 2,278). The restriction to the analysis period of 10 years is because the population of former sports soldiers could “only” be determined up to the year 2010 without gaps and thus statements on the representativeness of the sample for the years before 2010 would no longer have been reliably possible. Thus, the basic population (population 1) comprises *N* = 3,049 sports soldiers (Table 1).

**Table 1:** Sample and response rates.

	Population 1 (Basic population)	Population 2 (Contacted population)		Sample and response rates		
	*n*	*n*	%	*N*	% (Pop 1)	% (Pop 2)
Current	771	771	100.0	414	53.7	53.7
Former	2,278	1,973	86.7	298	13.1	15.1
Total	3,049	2,744	90.0	712	23.4	25.9

The current sports soldiers were contacted by the local management teams of the respective SPGs and sent a letter written by the research group and the German Armed Forces and the link to the online questionnaire. Since all current sports soldiers were contacted for participation, the gross and net populations for these cohorts are identical. The responses for the current sports soldiers were monitored for each SPG. The results were fed back to the German Armed Forces during the survey period. All current sports soldiers were then sent a reminder letter with reference to the questionnaire. If the response rate for a location was less than 20%, the leaders of the SPGs were then also contacted directly (sometimes several times) and asked to contact the current sports soldiers again and encourage them to participate. Due to data protection regulations (German Armed Forces), the former sports soldiers could not be contacted directly via the SPGs. They were instead primarily contacted (letter and link to the online questionnaire) by the relevant sports associations. The Olympic training centers and German sports aid program also helped with contacting the former sports soldiers. As not all former sports soldiers could be contacted, population 2 (contacted population) differs from population 1 (basic population). A total of *n* = 1,973 (population 2) of the former sports soldiers were contacted, meaning that 86.6% of population 1 (basic population) could be reached. Together with the 771 current sports soldiers, a total of *N* = 2,744 persons (population 2) were sent the link to the online questionnaire. The survey generated a sample of *n* = 414 current and *n* = 298 former sports soldiers. The net response rate (response to population 2) within the scope of the survey was 53.7% for current and 15.1% for former sports soldiers (Table 1). It is not surprising that the response rate among former sports soldiers is much lower than among current sports soldiers. On the one hand, unlike with the current sports soldiers, it was not possible for the SPG location leaders to contact them directly and encourage them to take part in the questionnaire. On the other hand, the connection to the SPGs is lower among the former sports soldiers. The SPGs are no longer employers for these cohorts and no longer serve as a central supporting authority for the (ended) sports career.

Women were slightly overrepresented in the sample among both current and former sports soldiers (Table 2).

**Table 2:** Representativeness of the sample in terms of gender.

	Population 1 (Basic population)		Population 2 (Contacted population)		Sample	
	*n*	%	*n*	%	*n*	%
Male						
Current	488	63.3	488	63.3	244	59.2
Former	1,695	74.4	1,441	73.0	205	69.3
Female						
Current	283	36.7	283	36.7	168	40.8
Former	583	25.6	532	27.0	91	30.7

Olympic sports were slightly underrepresented in the sample, with 87.5% (deviation −5.2%) compared to non-Olympic sports. Winter sports were slightly overrepresented in the sample, with 26.7% (deviation of +1.7%) compared to summer sports (Table 3).

**Table 3:** Representativeness of the sample in terms of sport (Olympic/non-Olympic and summer/winter).

	Population (Basic population)		Population 2 (Contacted population)		Sample	
	*n*	%	*n*	%	*n*	%
Sport status						
Olympic	2,827	92.7	2,545	92.7	623	87.5
Non-Olympic	212	7.0	189	6.9	86	12.1
Paralympic	5	0.2	5	0.2	3	0.4
Unassignable	5	0.2	5	0.2	0	0
Sport season						
Winter	763	25.0	665	24.2	191	26.7
Summer	2,281	74.8	2,074	75.6	517	72.8
Unassignable	5	0.2	5	0.2	4	0.5

### 3.2 Measurement

The current SWB was surveyed using the following question: *How satisfied are you currently with your life as a whole?* In addition, SWB in one year and in 5 years was queried as follows: *What do you think it will be like in one year? What do you think it will be like in 5 years*? The responses were collected on a 10-point scale from 1 (completely dissatisfied) to 10 (completely satisfied) as is common in surveys in the United Kingdom (92).

The independent variables were operationalized as follows: With regard to the *framework conditions of the SPG*, satisfaction with the services provided by the SPG was surveyed using four items (satisfaction with support for elite sports; satisfaction with support for professional future; satisfaction with career support service; satisfaction with medical support; *α* = 0.75). For this purpose, a 5-point scale from 1 (completely dissatisfied) to 5 (completely satisfied) was used, comparable to the measurement of satisfaction with the athletic career (7). The duration of membership (in years) in the SPG was also recorded.

For the *elite sport-specific factors*, the current squad status was queried for current sports soldiers and the highest squad status was queried for the former sports soldiers. For the further analyses, the Olympic and perspective squads were shown individually, while all other squads were combined into the category “other squads”. The information on the highest squad status of former sports soldiers can refer both to the old classification and to the new squad structures applicable as of January 1, 2018 (95). Therefore, the following recoding was carried out for the former sports soldiers whose squad classification is based on the old system (A squad = Olympic squad; B squad = perspective squad; all other squads = other squads). Four groups were formed to reflect sporting success (Olympic champion, Olympic medal, international medal at World or European Championships, no international medal). Satisfaction with the athletic career was surveyed as follows in accordance with Conzelmann and Nagel (7): *How satisfied are you currently with your sports career?* The answers were collected on a 5-point scale from 1 (completely dissatisfied) to 5 (completely satisfied). For current athletes, the average hours of training per week (13) and the number of injuries/illnesses in the last three years that led to a career interruption (training or competition absence) of more than three months (6) were also recorded, with this indicated dichotomously (break yes/no). In addition, on a 5-point scale from 1 (strongly disagree) to 5 (strongly agree), the former sports soldiers were asked how self-determined they felt the end of their career was: *The decision to end my top sports career was mainly due to external circumstances and less a voluntary decision*.

The educational status was recorded in the context of *socio-demographic and socio-economic factors*. A distinction was made between (i) those who had completed their studies, (ii) those who had completed their vocational training, (iii) those who were currently studying or undergoing training, and (iv) those who had neither completed their studies/training nor were currently studying/training. Current or former sports soldiers who were currently studying or undergoing vocational training but had already completed studies or training were assigned to the first or second group. The occupational status was also recorded for the former sports soldiers, whereby a distinction was made between (i) gainfully employed, (ii) not gainfully employed but in vocational training/studies, and (iii) not gainfully employed and not in vocational training/studies. Satisfaction with income was assessed using a 10-point scale from 1 (completely dissatisfied) to 10 (completely satisfied) (40). Furthermore, number of children and relationship status were queried in addition to age and gender. For the analysis, children (no, yes) and relationship status were operationalized dichotomously (single, committed relationship) (40).

The operationalization and description of the independent variables can be found in Table 4. With regard to variables relevant for both current and former sports soldiers, the following significant differences emerged: current sports soldiers showed slightly higher levels of satisfaction with the support services provided by the SPG (*M* = 4.03 vs. *M* = 3.83). The former sports soldiers had been with the SPG for a longer period (*M* = 4.94 vs. 6.70). The composition of the two groups in terms of squad status differed significantly in some cases. For example, the proportion of Olympic squads among former soldiers was about twice as high as among current soldiers (30.03% vs. 60.17%). This also applies to athletic success. In particular, the proportion of Olympic champions (1.98% vs. 5.31%) and medal winners at Olympic Games (1.70% vs. 4.42%) was significantly higher among former sports soldiers. A significantly higher proportion of former sports soldiers had completed their studies (15.58% vs. 54.87%) or vocational training (13.03% vs. 25.66%), while a higher proportion of current sports soldiers were still in vocational training/studies (37.68% vs. 10.18%) or were not in vocational training/studies and had not yet completed vocational training/studies (33.71% vs. 9.29%). On average, the former sports soldiers were significantly older than the current sports soldiers (*M* = 25.50 vs. *M* = 35.30). Female athletes were significantly more common among current sports soldiers (41.64% vs. 31.86%). The proportion of athletes who indicated single as their relationship status was almost twice as high among current sports soldiers (84.99% vs. 44.25%). The proportion of people with at least one child was significantly higher among former sports soldiers than current sports soldiers (7.08% vs. 52.65%).

**Table 4:** Description of the independent variables and testing for differences between current and former sports soldiers.

	Current sports soldiers (*n* = 353)			Former sports soldiers (*n* = 226)			Statistics
	%	M	SD	%	M	SD	
Satisfaction with SPG services (1 = completely dissatisfied to 5 = completely satisfied)		4.03	0.73		3.83	0.83	*U* = 45.668, *p* = .003, *r* = 0.123
Duration of membership SPG (years)		4.94	3.70		6.70	4.79	*U* = 32.892, *p* < .001, *r* = 0.148
Olympic squad	30.03			60.17			χ^2^ (2) = 55.967, *p* < .001, *V* = 0.311
Perspective squad	53.26			34.96			
Other squads	16.71			4.87			
Olympic winner	1.98			5.31			χ^2^ (3) = 10.227, *p* = .017, *V* = 0.133
Olympic medal	1.70			4.42			
International medal	57.79			49.56			
No international medal	38.53			40.71			
Satisfaction with sporting career (1 = completely dissatisfied to 5 = completely satisfied)		3.86	0.76		3.69	0.82	*U* = 44.517, *p* = .011, *r* = 0.106
Training hours per week		22.56	6.61		–	–	
Injuries/illnesses: No	81.59			–			
Injuries/illnesses: Yes	18.41			–			
Involuntary end of career (1 = strongly disagree to 5 = strongly agree)		–	–		2.29	1.59	
University degree	15.58				54.87		χ²(3) = 153.68, *p* < .001, *V* = 0.515
Completed vocational training	13.03.				25.66		
Currently studying or in vocational training	37.68				10.18		
No higher educational qualification	33.71				9.29		
Employed	–			86.73			
Not employed and in vocational training/studies	–			8.40			
Not employed	–			4.87			
Satisfaction with income (1 = completely dissatisfied to 10 = completely satisfied)		7.27	1.72		6.79	2.25	*U* = 43.635, (*p* = .053)
Age		25.50	4.08		35.30	5.99	*U* = 69.335, *p* < .001, *r* = 0.341
Female	41.64			31.86			χ²(1) = 5.201, *p* = .023, *V* = 0.098
Male	58.36			68.14			
Single	84.99			44.25			χ²(1) = 105.17, *p* < .001, *V* = 0.430
Committed relationship	15.01			55.75			
Children: No	92.92			47.35			χ²(1) = 150.72, *p* < .001, *V* = 0.514
Children: Yes	7.08			52.65			

### 3.3 Data analysis

The first step was to describe the current and future SWB of current and former sports soldiers. Multiple linear regressions (blockwise) were estimated in a second step to identify the factors that have a relevant influence on the SWB of the total study population. The blockwise approach allows the stability of the potential effects of individual factors to be assessed when additional factors are added to the model. As there is no homoscedasticity, robust standard errors were used (HC4) (96). The correlation is linear, and the residuals are normally distributed. Multicollinearity between individual explanatory variables was tested. No value higher than 3.89 was found for VIF (variance inflation factor). With a limit value of 10, there was therefore no multicollinearity (97).

## 4 Results

### 4.1 Descriptive analysis

Table 5 gives an overview of current and future SWB. The current (*M* = 7.64) and the former sports soldiers (*M* = 8.16) showed a high current well-being. Furthermore, both groups were optimistic about the future. The expected satisfaction value in one year was higher than the current value for both current (*M* = 8.16) and former sports soldiers (*M* = 8.37), and increased again with regard to the expected satisfaction value in 5 years (*M* = 8.57; 8.76). The former sports soldiers consistently showed significantly higher satisfaction values than the current sports soldiers, whereby the greatest difference were seen for current satisfaction.

**Table 5:** Current and future SWB of current and former sports soldiers.

	Current sports soldiers			Former sports soldiers			Statistics (Mann–Whitney-*U*-tests)
(10-point scale: 1 = completely dissatisfied, 10 = completely satisfied)							
	M	SD	*n*	M	SD	*n*	
Current SWB	7.64	1.63	353	8.16	1.66	226	*U* = 30,230, *p* < .001 *r* = 0.210
SWB in one year	8.16	1.62	392	8.37	1.65	289	*U* = 50,271, *p* = .009 *r* = 0.100
SWB in five years	8.57	1.74	376	8.76	1.63	284	*U* = 48,765, *p* = .046 *r* = 0.078

### 4.2 Multivariate analysis

#### 4.2.1 Model 1

By estimating a linear regression in model 1, the influence of the framework conditions of the SPG on the current SWB was examined (Tables 6, 7, column 1, each). For both current and former sports soldiers, a higher SWB was associated with greater satisfaction with the services offered by the SPG. The duration of membership in the SPG had no influence on SWB, neither for current nor for former sports soldiers.

**Table 6:** Factors influencing the current SWB of current sports soldiers [linear regression with robust standard errors, HC4, blockwise, displayed are non-standardized coefficients and standard deviation (in brackets); significance level: *p < .05; **p < .01; ***p < .001].

	Model 1	Model 2	Model 3 (overall model)
Framework conditions SPG			
Satisfaction with services SPG	0.294 [0.161]*	0.263 [0.157]*	0.197 [0.151]
Length of membership SPG	0.025 [0.027]	0.012 [0.031]	−0.018 [0.039]
Sport-specific factors			
Squad status (ref. Olympic squad)			
Perspective squad		−0.007 [0.213]	0.015 [0.212]
Other squads		0.152 [0.255]	0.164 [0.258]
Sporting success (ref. Olympic winner)			
Olympic medal		0.102 [0.196]	0.041 [0.193]
International medal		−0.449 [0.836]	−0.366 [0.893]
No international medal		−0.593 [0.971]	−0.685 [0.954]
Satisfaction with sporting career		0.727 [0.176]***	0.675 [0.189]***
Training hours per week		0.003 [0.013]	0.009 [0.013]
Injuries/illnesses (ref. no)			
Yes		0.018 [0.249]	−0.006 [0.241]
Socio-demographic & socio-economic factors			
Educational status (ref. university degree)			
Completed vocational training			0.100 [0.299]
Currently studying or in vocational training			−0.258 [0.265]
No higher educational qualification			−0.459 [0.285]
Satisfaction with income			0.166 [0.052]**
Age			0.025 [0.039]
Gender (ref. female)			
Male			0.350 [0.177]*
Relationship status (ref. single)			
Committed relationship			−0.044 [0.332]
Children (ref. no)			
Yes			−0.183 [0.453]
F	3.684*	5.287***	4.159***
*R*^2^	0.021	0.134	0.183
Adjusted *R*^2^	0.015	0.109	0.139
*N*	353	353	353

**Table 7:** Factors influencing the current SWB of former sports soldiers [linear regression with robust standard errors, HC4, blockwise, displayed are non-standardized coefficients and standard deviation (in brackets); significance level: *p < .05; **p < .01; ***p < .001].

	Model 1	Model 2	Model 3 (overall model)
Framework conditions SPG			
Satisfaction with services SPG	0.413 [0.178]*	0.403 [0.165]*	0.307 [0.151]*
Length of membership SPG	−0.019 [0.023]	−0.031 [0.026]	−0.032 [0.024]
Sport-specific factors			
Squad status (ref. Olympic squad)			
Perspective squad		−0.122 [0.266]	−0.052 [0.272]
Other squads		−0.455 [0.718]	−0.045 [0.721]
Sporting success (ref. Olympic winner)			
Olympic medal		−0.279 [0.266]	−0.134 [0.254]
International medal		−0.353 [0.491]	−0.341 [0.474]
No international medal		0.079 [0.854]	0.321 [0.396]
Satisfaction with sporting career		0.170 [0.405]	−0.013 [0.164]
Involuntary end of career		−0.187 [0.075]*	−0.161 [0.076]*
Socio-demographic & socio-economic factors			
Educational status (ref. university degree)			
Completed vocational training			−0.297 [0.292]
Currently studying or in vocational training			−0.705 [0.516]
No higher educational qualification			0.081 [0.349]
Professional status (ref. employed)			
Not employed and in vocational training/studies			0.860 [0.505]
Not employed			−0.092 [0.586]
Satisfaction with income			0.237 [0.054]***
Age			0.009 [0.021]
Gender (ref. female)			
Male			−0.174 [0.238]
Relationship status (ref. single)			
Committed relationship			0.485 [0.256]*
Children (ref. no)			
Yes			0.137 [0.242]
F	4.649*	2.535**	3.554***
*R*^2^	0.040	0.096	0.247
Adjusted *R*^2^	0.031	0.058	0.177
*N*	226	226	226

#### 4.2.2 Model 2

When sport-specific factors are integrated (Model 2, see Tables 6, 7, column 2 for each), the effect shown in Model 1 regarding satisfaction with the services of the SPG remains stable for current and former sports soldiers. In addition, satisfaction with athletic career showed a positive effect on SWB for current sports soldiers. This influence was not shown for former sports soldiers. Among former sports soldiers, a career end that was perceived as rather involuntary had a negative effect on SWB. In the model of both current and former sports soldiers, athletic success and squad membership had no influence on SWB. Current sports soldiers also showed no effect for amount of training or career interruptions due to injuries/illness.

#### 4.2.3 Model 3 (full model)

Taking socio-demographic and socio-economic factors into account (Model 3, see Tables 6, 7, column 3), the positive effect of satisfaction with the performance of the SPGs in Models 1 and 2 decreased among current sports soldiers and was no longer significant. In contrast, the effect remains stable among former sports soldiers. The influence of satisfaction with the sports career among current sports soldiers remained unchanged, as did the influence of involuntary end of career among former sports soldiers. With regard to the socio-demographic and socio-economic factors, higher satisfaction with income goes hand in hand with higher satisfaction values in relation to SWB for both current and former sports soldiers. Higher satisfaction scores were reported by current male sports soldiers. Furthermore, former sports soldiers in a committed relationship showed higher SWB.

The quality in model 1 (current sports soldiers: adjusted *R*^2^ = .015; former sports soldiers: adjusted *R*^2^ = .031) was improved by including sport-specific factors in model 2 (current sports soldiers: adjusted *R*^2^ = .109; former sports soldiers: adjusted *R*^2^ = .058) and socio-demographic/socio-economic factors into the overall model 3 (current sports soldiers: adjusted *R*^2^ = .139; former sports soldiers: adjusted *R*^2^ = .177).

## 5 Discussion

### 5.1 Contribution to the literature and discussion of the results

The present study follows calls for a more athlete-centered approach in sports science research (98–102). The present study extends the existing body of research on elite sports development in specific settings, such as the military context in general and with regard to SWB in particular. A special feature of the present study is that both current and former elite athletes are included in the analysis. By examining both cohort-specific and overarching factors for their influence on SWB, the relevance of these factors in the respective life phases can be (comparatively) analyzed. In addition, the influence of funding structures that change over time can be mapped. Furthermore, this study focuses on specific framework conditions, life circumstances, as well as factors in the context of individual self-realization, which enables a differentiated and context-related analysis.

The present study generated the following central findings: The current SWB of current sports soldiers (*M* = 7.64) is at a similarly high level as that of elite athletes in Germany in general (*M* = 7.46), but also in relation to the general population (*M* = 7.55) (40). This also applies to former sports soldiers (*M* = 8.16), who have a similarly high SWB as former professional soccer players in Germany (103). Due to restriction of data structure (cross sectional data) it is not possible to estimate with any certainty to what extent SWB of the current and former sports soldiers was causal (negatively) influenced by the COVID-19 pandemic at the time of conducting the survey. The available findings do not indicate any significant influence. It should be noted that, as they are employed by the German Armed Forces, the current sports soldiers also had financial security during the COVID-19 pandemic. It is therefore unlikely that financial concerns had a negative impact on the SWB of current sports soldiers during the COVID-19 pandemic. Moreover, both current and former sports soldiers are optimistic about the future. If we also consider that the SWB in the present study was surveyed on a 10-point scale and not on an 11-point scale as in the comparative studies, then the reported SWB values even represent rather conservative estimates.

Higher satisfaction with the benefits of the SPG as an employer is associated with higher SWB values among current sports soldiers. This is consistent with findings from general SWB research, which have shown that job satisfaction is positively related to SWB (66–68). However, in the overall model, the influence of satisfaction with the performance of the SPG is no longer significant, but is overshadowed by the strong influence of financial satisfaction. For former sports soldiers, higher satisfaction values with the services of the SPG go hand in hand with higher SWB in all models. Although the SPG was no longer the current employer for the former sports soldiers, the long and important involvement in the SPG appears to have an impact on the SWB of many athletes. However, it is also conceivable that both factors correlate with each other but are not causally related.

The fact that the SWB of current sports soldiers is influenced by satisfaction with their athletic career can be explained by the strict orientation towards success in elite sports (69). No influence was shown in this regard for former sports soldiers who had already ended their careers. However, in the case of former sports soldiers, the perceived involuntary career end of their elite athletic career had a negative influence on SWB, which is consistent with previous studies (41, 44). Comparable to the findings by Wicker et al. (40), in which only Olympic champions showed slightly higher SWB, athletic success (squad status, international medals) had no effect in the present study. Surprisingly, however, among current sports soldiers, the presence of injuries/illnesses that prevented them from participating in training or competitions for more than three months in the last three years had no influence on SWB, which contrasts with previous findings (72). It can be assumed that the lack of influence is related to the operationalization of the presence of injuries/illnesses in the present study. The factor was only recorded dichotomously, but a comparatively long period of time was chosen with the retrospective view of the last three years, so that injuries/illnesses that occurred further in the past would no longer influence the current SWB. The cancellation of competitions during the COVID-19 pandemic could also have meant that injuries/illnesses had no impact on the athletes' SWB.

Regarding socio-demographic & socio-economic factors, it is clear that higher satisfaction with income is associated with higher SWB among both current and former athletes, which confirms previous findings in the context of elite sports (40) as well as in other settings (75, 78, 79, 88, 89). A secure financial situation or high level of satisfaction with current income is a decisive factor for both current and former athletes with regard to SWB. The influence of gender was inconsistent. While gender had no influence on SWB in the former sports soldiers, the current male sports soldiers showed higher satisfaction values than the female ones, contrary to the study situation (75, 79, 87, 89–91). The extent to which this finding, which has already been observed in isolated cases in other contexts (78, 86), is related to the specific setting of elite sports cannot be answered based on the available data. The finding that former sports soldiers who are in a committed relationship have higher satisfaction scores is consistent with findings from previous studies (75, 78, 79, 88–90, 92). There was no effect in this respect for current sports soldiers. However, almost 85% of the current sports soldiers stated that they were single.

There were no significant findings with regard to professional status. There was a tendency (*p* < 0.1) for former sports soldiers who were not employed but were pursuing vocational training or higher education to show the highest SWB. One explanation for this may be that the possibility of more flexible time management leads to higher SWB values, particularly for students. Especially among former elite athletes whose lives were characterized by clear guidelines for many years, the perception of greater self-determination can increase satisfaction. Contrary to the assumption (75–79), education-related factors had no influence on SWB for current or for former sports soldiers. One possible explanation could be that the overwhelming proportion of elite athletes have school-leaving qualifications that entitle them to higher education (104), which makes it difficult to draw sufficient contrasts in terms of educational status.

### 5.2 Limitations and future research

It is important to consider not only the strengths of the present study, but also its limitations: (i) The inclusion of the athletes was based on the premise that they were currently members of an SPG or had left an SPG in the past ten years. This procedure is appropriate for the objective of the contribution. However, since the quotas (number of SPG places) vary greatly between sports, there are large differences in the size of the sport-specific samples. In addition, only very small samples could be generated for individual sports, which is why a differentiated analysis by sport was not possible. In further studies, this should be taken into account to be able to analyze possible sport-specific differences in SWB. (ii) It should be noted that the representative nature of the sample in terms of gender, winter sports vs. summer sports and Olympic sports vs. non-Olympic sports is satisfactory. However, a non-response bias that could lead to the SWB overestimated cannot be excluded. This particularly applies to former sports soldiers, who demonstrate a significantly lower response rate than current sports soldiers. It is therefore recommended to place the focus on former elite athletes in further investigations (iii) By including satisfaction with the support structures in the SPG, the influence of a specific setting on SWB was analyzed. Future studies should expand the view to other networks and support services (39, 105) that are relevant for athletes to achieve peak performance and to examine their influence on SWB. (iv) Several sport-specific factors were considered in the present study. However, the operationalization of the factor injuries/illnesses is associated with clear limitations. Against the background of the strong reference to the body, injuries/illnesses in elite sports represent a central challenge (3, 6, 106), which is why this factor should be mapped in a more differentiated way in further studies on SWB of elite athletes. (v) For former elite athletes, the transition to a professional career is an important point in their lives (10, 107). The influence of profession on SWB should therefore be analyzed in a more differentiated way in further studies. For example, satisfaction with the current occupation should be integrated into the analysis. (vi) By considering both factors of the environment/living circumstances and factors within the framework of individual self-realization, the present paper aims at a holistic analysis (60) including factors within and outside a person. However, the focus in the present study is on external factors, so that future studies should emphasize an even more balanced inclusion of both perspectives. The inclusion of psychological concepts, such as self-worth, self-perception, or self-esteem, is recommended (108, 109).

### 5.3 Practical implications and awareness-raising measures

The following conclusions for organized sports can be drawn from the findings: (i) The SPG represents a specific model of support for elite sports. It has been shown that satisfaction with the services offered in this setting has a direct influence on SWB. It is therefore useful for organized sports to evaluate which services the elite athletes are satisfied with and which services they are dissatisfied with to optimize the latter. This would not “only” increase the SWB of sports soldiers, but also have a positive influence on elite sports performance. (ii) With regard to factors specific to elite sports, how an athletic career ends proves to be an important factor for SWB, even after the transition phase into the post-sports career. In order to prevent top athletes from perceiving the end of their career as involuntary, organized sports should be transparent in communicating performance expectations and opportunities so that athletes can prepare themselves early for a career end due to a decline in performance. Furthermore, since elite sports careers can end involuntarily due to injuries, organized sports should anchor structures for injury management in clubs and federations, which also includes prevention measures or dealing with presenteeism (3, 6). (iii) Even though the findings were not significant with regard to professional status, they tend to show that former athletes who pursue vocational training have higher satisfaction scores. For organized sports, this results in the need to support opportunities for vocational qualification during, but also after, the end of a professional sports career. The transition payments from the German Armed Forces (temporary financial support for the implementation of professional training after the end of membership in the SPG) represent an important element here. (iv) The SWB of elite athletes is influenced largely by financial satisfaction. Satisfaction with income proves to be an important factor influencing SWB across studies. Sports soldiers are financially secure within the support structures of the German Armed Forces, which means that sports soldiers have higher satisfaction values than other athletes. Nevertheless, there are differences between the sports soldiers with regard to satisfaction with income, which in turn has an impact on SWB. This is because the opportunities to generate income through elite sports itself differ considerably between sports. Athletes earn comparatively low incomes, especially in sports that receive little television coverage (110). In addition, especially in sports with good earning opportunities for male athletes, there are sometimes considerable gender-specific income differences (111). As a result, top athletes in Germany who are not involved in state-related support structures, such as the SPG of the German Armed Forces, often have an imputed average hourly wage that is below the legal minimum wage (13). Based on this, organized sports should focus on adequate financial support to increase athlete satisfaction.

## Data Availability

The datasets presented in this article are not readily available because the dataset is restricted due to sensitive data. Requests to access the datasets should be directed to pehnold@ist-hochschule.de.
